# First Definition of Reference Intervals of Liver Function Tests in China: A Large-Population-Based Multi-Center Study about Healthy Adults

**DOI:** 10.1371/journal.pone.0072916

**Published:** 2013-09-13

**Authors:** Runqing Mu, Wenxiang Chen, Baishen Pan, Lanlan Wang, Xiaoke Hao, Xianzhang Huang, Rui Qiao, Min Zhao, Chuanbao Zhang, Wei Guo, Hengjian Huang, Yueyun Ma, Junhua Zhuang, Jie Zhang, Hong Shang

**Affiliations:** 1 Department of Laboratory Medicine, The First Hospital of China Medical University, Shenyang, China; 2 National Center for Clinical Laboratories, Beijing Hospital, Beijing, China; 3 Department of Laboratory Medicine, Zhongshan Hospital of Fudan University, Shanghai, China; 4 Department of Laboratory Medicine, West China Hospital, Sichuan University, Sichuan, China; 5 Department of Laboratory Medicine, Xijing Hospital, The Fourth Military Medical University, Xi'an, Shaanxi, China; 6 Department of Laboratory Science, The Second Affiliated Hospital of Guangzhou University of Chinese Medicine, Guangdong, China; 7 Department of Laboratory Medicine, Peking University Third Hospital, Beijing, China; Yonsei University College of Medicine, Korea, Republic of

## Abstract

**Background:**

Reference intervals of Liver function tests are very important for the screening, diagnosis, treatment, and monitoring of liver diseases. We aim to establish common reference intervals of liver function tests specifically for the Chinese adult population.

**Methods:**

A total of 3210 individuals (20–79 years) were enrolled in six representative geographical regions in China. Analytes of ALT, AST, GGT, ALP, total protein, albumin and total bilirubin were measured using three analytical systems mainly used in China. The newly established reference intervals were based on the results of traceability or multiple systems, and then validated in 21 large hospitals located nationwide qualified by the National External Quality Assessment (EQA) of China.

**Results:**

We had been established reference intervals of the seven liver function tests for the Chinese adult population and found there were apparent variances of reference values for the variables for partitioning analysis such as gender(ALT, GGT, total bilirubin), age(ALP, albumin) and region(total protein). More than 86% of the 21 laboratories passed the validation in all subgroup of reference intervals and overall about 95.3% to 98.8% of the 1220 validation results fell within the range of the new reference interval for all liver function tests. In comparison with the currently recommended reference intervals in China, the single side observed proportions of out of range of reference values from our study for most of the tests deviated significantly from the nominal 2.5% such as total bilirubin (15.2%), ALP (0.2%), albumin (0.0%). Most of reference intervals in our study were obviously different from that of other races.

**Conclusion:**

These used reference intervals are no longer applicable for the current Chinese population. We have established common reference intervals of liver function tests that are defined specifically for Chinese population and can be universally used among EQA-approved laboratories located all over China.

## Introduction

Liver function tests, including assays for alanine aminotransferase (ALT), aspartate aminotransferase (AST), alkaline phosphatase (ALP), γ-glutamyltansferase (GGT), total protein, albumin, and total bilirubin, are generally used to assess hepatocellular injury, cholestasis, infiltrative disease, biliary obstruction, or synthetic function of the liver. Normally, liver function tests are also used to screen asymptomatic patients/individuals, mostly during regular health check-ups, blood donation, and hospitalization for non-liver related diseases [1]. Appropriate reference intervals of those tests are the most important elements for health evaluation, disease diagnosis, therapy monitoring, and prognosis assessment.

Currently, Chinese laboratory reference intervals are mostly derived from the National guide to Clinical Laboratory Procedures (third edition) [2] which addresses the currently recommended reference intervals in China from various sources such as books, manuscripts, and sometimes manuals. Part of the reference intervals are from the user instructions and reagent application manuals provided by manufacturers. In most cases these values are based on more than a decade old studies in European and American populations. In some labs, reference intervals in use are self-established by testing local healthy volunteers [3]. As a consequence, they vary from each other significantly among routine diagnostic laboratories across China. For example, the upper limits of the ALT reference intervals range from 25 to 152 U/L [3]. In China, the quality of available medical care is largely region dependent. This leads to a very high patients' mobility across regions, as most patients tend to go to larger hospitals or hospitals with higher medical standards [4]. With patients moving between different hospitals, it is essential to establish and apply common reference intervals for the Chinese population.

With the increasing degree of standardization of laboratory, members of the scientific community are now proposing that it is now feasible to establish common reference intervals [5]. For example, the international community has established common reference intervals through several multi-center studies, based on the populations of five Nordic countries, seven Southeastern African countries and multiple ethnic groups from different countries respectively [6], [7], [8]. However, it is not appropriate to directly apply these reference intervals to the Chinese population, because some parameters may vary significantly among different races [8], [9], [10]. Moreover, currently there are no large sample multi-center based reference intervals developed for the Chinese population. Recently, several studies described the reference intervals established using specific testing systems for the Chinese populations of specific locations, such as Hong Kong [11] and Beijing [12]. Nevertheless, taking into account the large diversity of the Chinese population in age structure, geographical, nutritional and occupational status, these local reference intervals cannot be applied to the whole Chinese population. Therefore, it is necessary to establish liver function tests' reference intervals which are specific for the Chinese population and can be universally applied in most hospitals across China.

In order to achieve this goal, we designed a multi-center study with a large number of reference individuals recruited from all six main geographic regions of China to establish reference intervals specific and appropriate for the Chinese population in accordance to the C28-A3 guideline from Clinical and Laboratory Standards Institute (CLSI) [13]. Furthermore, we also designed a validation study to further evaluate the national applicability of the newly established reference intervals. We expect that they can be directly applied to most Chinese people living in China and may also be considered for the Chinese populations living abroad. Moreover, our protocols may be helpful to other countries with similar population structure and medical situation.

## Methods

### Ethical Considerations

This study was approved by the ethics committee of the First Hospital of China Medical University. All reference individuals enrolled in this study written informed consent prior to the study.

### Selection of reference population

#### Source of population

**Figure 1:**
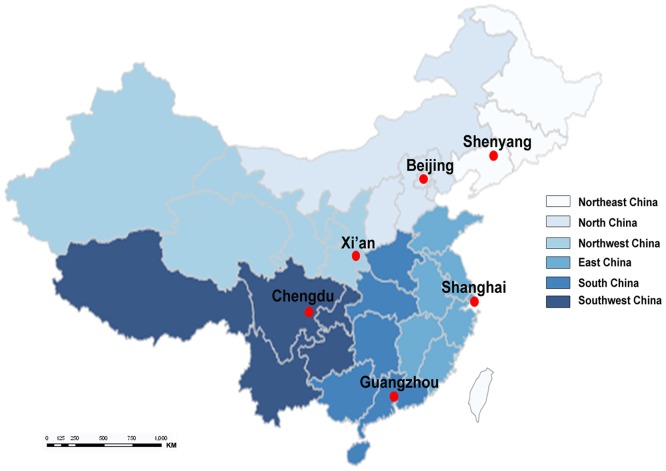
Map of six clinical research centers in this study.

**Figure 2:**
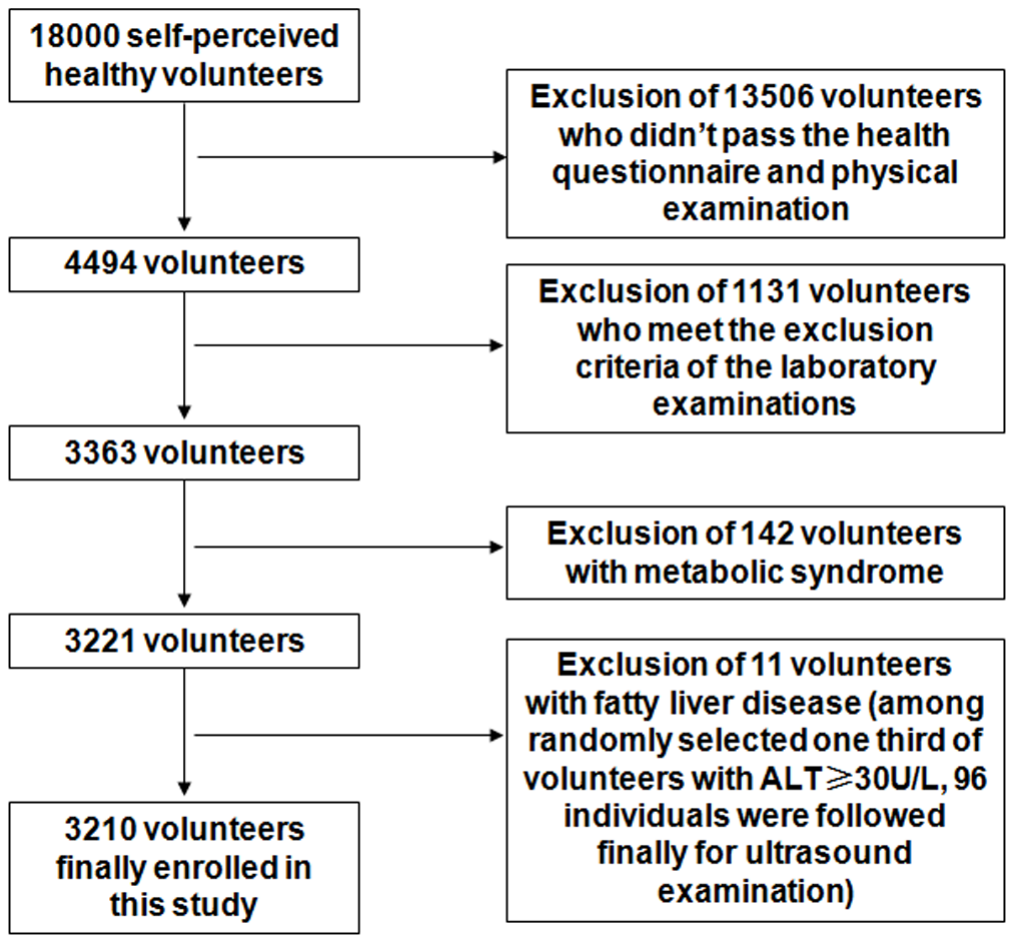
Procedures for selection of the study participants.

A multi-level cluster sampling approach was used to recruit apparently healthy individuals (taken into account factors such as gender, age, region, and urban-rural) from the six representative geographical regions in China: Northeast China (Shenyang), North China (Beijing), East China (Shanghai), South China (Guangzhou), Southwest China (Chengdu) and Northwest China (Xi'an) (Figure 1). For this study, the target population was all Han Chinese (account for 91.6% of the total Chinese population [14]). With age ranged from 20 to 79 years, 3210 adults met the exclusion criteria finally. Figure 2 summarizes the selection of the study participants.

#### Exclusion criteria

For the recruited self-perceived healthy individuals, each candidate was required to complete a questionnaire and a physical examination by a physician to check their health conditions. The exclusion criteria were as followings: presence of acute and chronic infections, digestive diseases, kidney disease, metabolic and nutritional diseases, rheumatic diseases, endocrine disease, circulation system diseases, burns and muscle trauma, hypertension (systolic pressure ≥140 mmHg and/or diastolic pressure ≥90 mmHg), body mass index (BMI)≥25 kg/m^2^, excessive alcohol consumption (alcohol consumption>30 g/day), excessive smoking (smoking>20 cigarettes/day), massive blood loss, malnutrition (with clear causes (lose weight, poverty, or special dietary habits) and symptoms (low BMI or significant weight loss) of malnutrition), surgery undergone within six months, medication taken within two weeks, blood donation or blood transfusion within four months, strenuous exercise or heavy manual labor, women in pregnancy or lactation.

Individuals are further excluded in accordance with one of the following criteria: Positive results for Hepatitis B surface antigen, Hepatitis C antibodies, or HIV antibodies; Exceeding one or more cut-off thresholds shown in Table 1; having metabolic syndrome (MS) [15]. In addition, among the 339 finally qualified individuals with ALT≥30 U/L, we randomly selected about one third (96 of 113 selected volunteers finally participated) for ultrasound examination to evaluate the influences of fatty liver disease (FLD) on establishing the reference intervals. Eleven volunteers with ultrasound-diagnosed FLD were excluded from the study.

**Table 1:** Analytes and the corresponding thresholds for exclusion of individuals.

Analytes	Threshold
Creatinine	>120 µmol/L
Creatine kinase	>500 U/L
Uric acid	>475 µmol/L
Albumin	<25 g/L
Triglycerides	≥2.26 mmol/L
Total cholesterol	≥6.22 mmol/L
White blood cells	>12.5×10^9^/L or <3.0×10^9^/L
Hemoglobin	<120 g/L(males)
	<110 g/L(females)
Fasting glucose	≥7.0 mmol/L

### Laboratory analysis

#### Sample collection and handling

Participants were informed that during the last three days prior to sample collection, they should keep their living and diet habits as usual but should avoid alcohol-intake during the last 24 hours and smoking during 1 hour prior to blood sample collection. Individuals were required to donate blood samples in the morning, with a fasting time more than 8 hours but less than 14 hours. The blood samples were collected from the cubital vein and dispensed into 5 ml SST tube with gel (Becton Dickinson, USA). All blood samples were centrifuged to separate serum at 1200 g for 10 minutes within 2 hours after collection and serum was analyzed within 2 hours after separation.

#### Assay of screening tests

Hepatitis B surface antigen (Wantai Biological Pharmacy, China), Hepatitis C antibodies (Kehua Bio-engineering, China), HIV antibodies (Livzon diagnostics, China) were tested using ELISA method. Hematology tests were performed using Sysmex XE2100 hematology analyzers (Sysmex, Japan). Chemistry tests were assayed by Roche Modular automatic biochemical analyzer (Roche, Germany).

#### Assay of liver function tests

Roche Modular automatic biochemical analyzer (Roche, Germany), Beckman DXC800 automatic biochemical analyzer (Beckman Coulter, USA) and Olympus AU680 automatic biochemical analyzer (Beckman Coulter, USA) were used for assaying the concentration of liver function tests because these systems are mainly used by clinical laboratory in China currently. Reagents and calibrators are from the corresponding manufacturer of instrument. Roche Modular analyzers were used in all six study centers, in addition Beckman DXC800 analyzers were used in the centers of Shenyang and Chengdu, and Olympus AU680 analyzers were used in the centers of Shenyang and Beijing. All tests were performed on the samples collected locally according to the instrument Operating Instructions of the manufacturer.

For trueness assessment: traceability is to demonstrate how a measurement result can be traced to stated national or international standards through an unbroken chain. This study includes the trueness assessment for the validation of traceability so that we can evaluate whether or not the testing results can be traced to reference materials and reference measurement methods/procedures provided by JCTLM (Joint Committee on Traceability in Laboratory Medicine). Prior to analyzing the samples from the reference collective, we first assessed the analytical trueness of the participated laboratories: certified reference materials were tested using the three different measuring systems in all six study centers, five times in a row per day, for two days. The regular trueness assessment was performed during the formal testing period by testing the certified reference materials every month. For each time of the assessment, the reference materials were tested five times within the same lot and the average test values of reference materials were calculated. The certified reference materials include: (1) China national standard materials (base material is pooled human serum and values are assigned using the reference method recommended by JCTLM): ALT, AST, GGT, total protein (three levels), total bilirubin (two levels); (2) CRM470 (NIST, USA): albumin (one level). Trueness expressed by relative bias between the means of measured values of reference materials obtained by the analytical system and the assigned target values of reference materials. To pass the assessment, trueness should meet the desirable quality specification of biologic variations on the inaccuracy [16].

Currently, the International recommended reference methods for ALT and AST are using the reagents with pyridoxal-5′-phosphate. The test results of analytical reagents with pyridoxal-5′-phosphate are identified as ALTp, ASTp which can perform trueness assessment, and the test results of analytical reagents without pyridoxal-5′-phosphate are identified as ALTnp, ASTnp in our study. The latter reagent are used in most laboratories in China, therefore the results using two kinds of reagents were both stated in this study for ALT and AST.

For precision assessment: all participating laboratories were required to use the approved commercial quality control materials which should consist of both normal and abnormal levels and are produced from the same manufacturer with the same lot number. During the time prior to formal testing, the laboratories should repetitively test one set of these quality control materials for four times per day for five days. During sample analysis stage, two commercial controls and an blinded frozen human serum sample was tested with each analytical batch for assessment of the quality of routine laboratory testing and the consistency of test results between different laboratories. The precision should meet the desirable quality specification of biologic variations on the imprecision [16].

#### Processing of test results

Results obtained by measurement of reference individuals were directly used for establishment of reference intervals, if the relative bias between the means of measured values of reference materials obtained by the analytical systems and the assigned target values of reference materials meet the desirable specification for inaccuracy of biologic variation. In cases where the relative bias exceeds the quality specifications: a re-standardization has been applied: if the reference materials are of more than one concentration levels, a linear regression equation was established based on the means of reference materials and the assigned target values of the reference materials and the results of the samples from the reference collective are adjusted in accordance with the established linear regression equation; if the reference materials are of only one concentration level, the correction factors were calculated and applied directly to adjust the relevant results of the samples.

### Data Analysis

The statistical analysis of the data in this study was performed using JMP 9.0, SAS 9.1 and SPSS 17.0.

#### Establishment of reference Intervals

Partitioning of reference Values was determined based on the test results from Roche system. We applied a 3-level nested ANOVA to evaluate the importance of gender, age, and region as sources of variation in the multi-center study and then separated the magnitude of variations attributable to each factor and expressed it in terms of the SD. It computes the SD ratio (SDR), which is the SD of a given factor divided by the SD due to between-individual variation. For the factor with SDR less than 0.3 a partitioning was considered as not necessary [17], [18]. Instead, we need further analysis: in this case reference values were divided into different subgroup by one-way ANOVA for partitioning variable.

Outlier data exclusion: (1) For enzyme assays, if the data is skewed, datapoints lying outside the interval of mean ± 4sd were excluded after logarithm transformation [6]; (2) For non-enzyme assays (total protein, albumin, and total bilirubin), Dixon's rule was applied for outlier determination [13]. The reference interval was computed using nonparametric method after excluding the outliers. The lower and upper reference limits were defined as 2.5^th^ and 97.5^th^ percentiles of the distribution of values respectively, and 90% confidence limits were computed by nonparametric method for each reference limit as well. Since the albumin level shows a strong age-dependence, stratified random sampling method was used to introduce age groups matching the age distribution from the 6th nation-wide census of Chinese population in 2010 [14]. On that basis age-standardized albumin reference interval is calculated.

#### Comparison of analytical system

One-way ANOVA was used to compare the differences of results obtained from different instruments.

#### Validation of reference intervals

**Figure 3:**
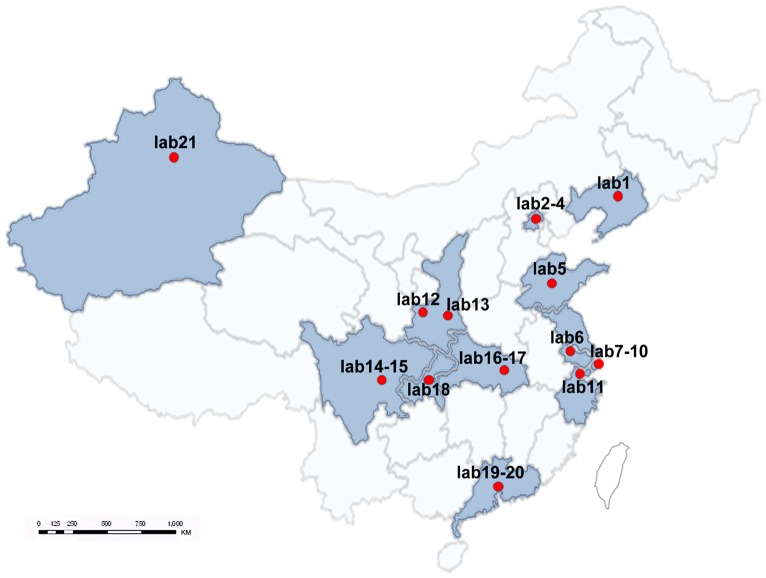
Map of 21 laboratories in China selected to perform reference interval validation.

For the newly established reference intervals, which are based on population living in the six regions of China using three analytical systems, we need to assess applicability for different testing systems/conditions and the Chinese sub-population living across the whole country. For this purpose, we referred to CLSI C28-A3 guideline and performed the validation for the newly established reference intervals as stated there. We selected 21 large hospitals located in 12 provinces and cities nationwide including Lab 1(Liaoning), Lab 2∼4(Beijing), Lab 5(Shandong), Lab 6(Jiangsu), Lab 7∼10(Shanghai), Lab 11(Zhejiang), Lab 12∼13(Shaanxi), Lab 14∼15(Sichuan), Lab 16∼17(Hubei), Lab 18(Chongqing), Lab 19∼20(Guangdong), and Lab 21(Xinjiang Autonomous Region) (Figure 3). These laboratories were qualified by the National External Quality Assessment of China and selected to perform the reference interval validation. For each subgroup of reference intervals, 20 subjects that met the exclusion criteria of the study protocol were recruited locally for each of the 21 selected hospitals. Routine instruments, reagents and controls were used to test the samples of the locally recruited individuals in each laboratory including open-system (instruments, reagents and calibrators are not produced from the same manufacturer) and close system (analytical system recommended by the manufacturer). The 20 test results of these individuals were evaluated with the newly established reference intervals. If no more than two out of the 20 subjects' exhibit results(or 10% of the test results) outside the limits then the newly established reference interval was considered to be valid for the sub-population [13].

## Results

### Study population

A total of 3210 individuals were enrolled in six regions with 1274 males, 1936 females, and a male∶ female ratio of 1∶1.52. Among these individuals, 2102(65.5%) were from urban areas and 1108(34.5%) were from rural areas (Table 2). The study cohort consists of subjects with different educational and economical background. 16 occupations such as instructor, student, worker, medical staff, government officer, corporation employee, farmer, etc were included. There were at least 400 subjects in each region or age subgroup. Demographically, among the 6 regions the mean age of the subjects recruited in Guangzhou area was significantly higher than that of other regions (*P*<0.05), whereas the mean age of the subjects from Xi'an area was significantly lower than that of the other regions (*P*<0.05).

**Table 2:** Characteristics of the reference population enrolled in this study.

Variable		Total	Shenyang	Beijing	Shanghai	Guangzhou	Chengdu	Xi'an
Qualitative variables		n(%)	n(%)	n(%)	n(%)	n(%)	n(%)	n(%)
Total		3210	489(15.2)	472(14.7)	431(13.4)	742(23.1)	551(17.2)	525(16.4)
Gender	Male	1274(42.4)	173(35.4)	202(42.8)	163(37.8)	291(39.2)	220(39.9)	225(42.9)
	Female	1936(57.6)	316(64.6)	270(57.2)	268(62.2)	451(60.8)	331(60.1)	300(57.1)
Area	Urban	2102(65.5)	363(74.2)	327(69.3)	308(71.5)	459(61.9)	300(54.5)	345(65.7)
	Rural	1108(34.5)	126(25.8)	145(30.7)	123(28.5)	283(38.1)	251(45.6)	180(34.3)
Age group	20–29	990(30.8)	144(29.5)	162(34.3)	127(29.5)	188(25.3)	158(28.7)	211(40.2)
	30–39	673(21.0)	89(18.2)	104(22.0)	99(23.0)	126(17.0)	125(22.7)	130(24.8)
	40–49	615(19.2)	131(26.8)	79(16.7)	67(15.6)	123(16.6)	126(22.9)	89(17.0)
	50–59	487(15.2)	79(16.2)	63(13.4)	87(20.2)	101(13.6)	90(16.3)	67(12.8)
	60–79	445(13.9)	46(9.4)	64(13.6)	51(11.8)	204(27.5)	52(9.4)	28(5.3)

Quantitative variables		Mean±SD	Mean±SD	Mean±SD	Mean±SD	Mean±SD	Mean±SD	Mean±SD
Age	year	40.6±15.3	40.1±14.0	39.3±15.6	40.6±14.3	45.7±17.7	40.3±13.4	35.6±13.0
BMI	kg/m^2^	21.5±2.0	21.7±1.8	21.8±1.9	21.5±2.0	21.1±2.1	21.6±2.0	21.6±1.9
Triglycerides	mmol/L	0.94±0.38	0.88±0.35	0.91±0.37	0.94±0.41	0.97±0.38	0.93±0.37	0.95±0.39
Total cholesterol	mmol/L	4.64±0.76	4.72±0.73	4.66±0.74	4.59±0.75	4.82±0.73	4.71±0.72	4.27±0.75
Glucose	mmol/L	5.02±0.45	4.93±0.43	4.92±0.46	4.92±0.41	5.06±0.48	5.03±0.43	5.21±0.39

### Reference intervals of liver function tests of adult population in China

**Figure 4:**
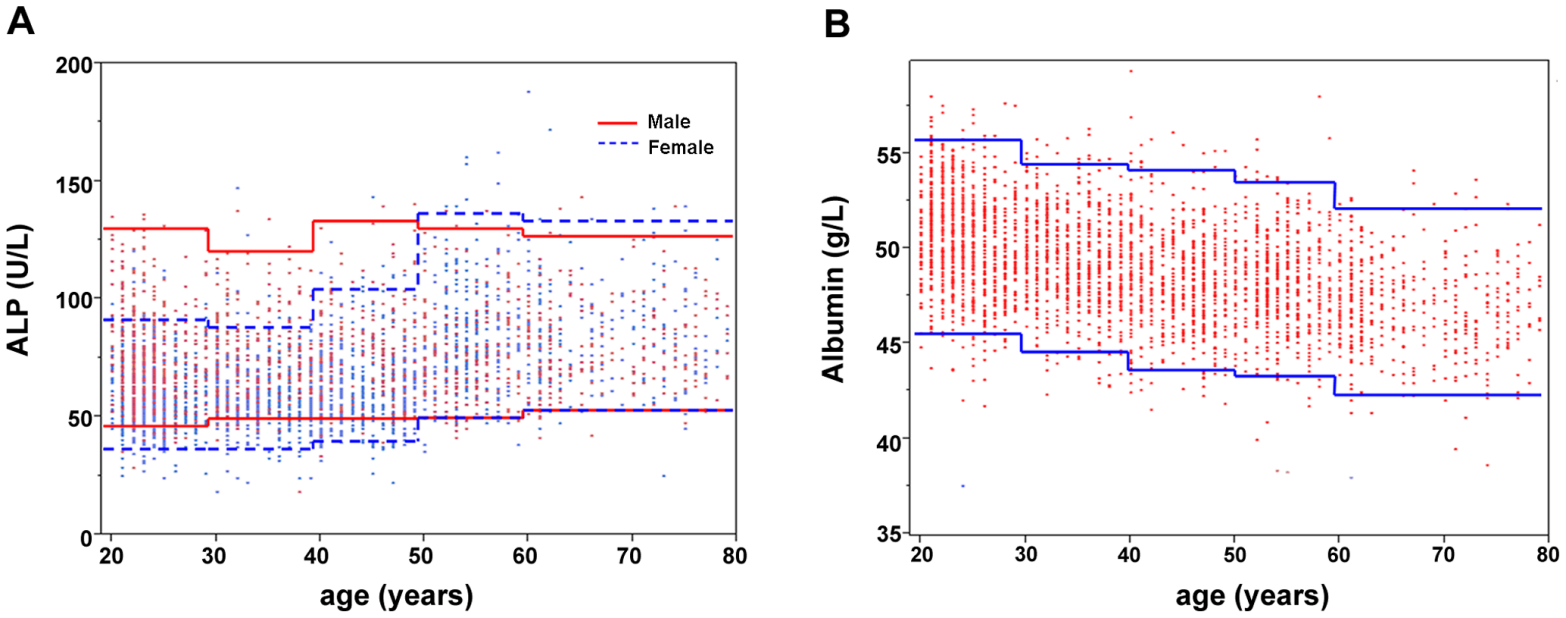
The scatterplots of ALP and albumin distributions as a function of age. The lines represent the lower (2.5^th^) and upper (97.5^th^) reference limits for different age groups in the scatterplots of ALP(A) and albumin(B) with age.

Table 3 summarizes the reference intervals of liver function tests of the adult population in China. The reference intervals of ALTp, ASTp, total protein, and total bilirubin were based on the test results with traceability. The reference intervals of ALTnp, ASTnp, ALP, albumin, for which traceability cannot be confirmed were based on the test results from Roche instruments. The study shows a strong gender-dependence of the reference intervals for ALT, GGT, and total bilirubin with significantly higher values in males than in females. There were also apparent age variations of the reference intervals for albumin and ALP. As shown in Figure 4, albumin level gradually decreases with aging (SDR = 0.51). No difference was observed between the age-standardized albumin reference interval (43.4–54.5 g/L) and those without age standardization (43.4–54.6 g/L), so there was no sampling bias for albumin in this study. The dependency of ALP reference interval on age is different for males and females (Figure 4). For females of aged 50 or more there was a significant increase (SDR = 0.74). In contrast, there was no significant age dependence in males (SDR = 0.05). Thus, the ALP reference values were partitioned by gender, and the age of 50 was used as the cutoff for females. The result of total protein had significant regional difference (SDR = 0.36). Further analysis indicates that total protein levels in Shenyang and Shanghai areas were significantly higher than those in other areas by multiple comparison of one-way ANOVA.

**Table 3:** Reference intervals of liver function tests of the adult population in China.

Analytes	Unit	Partition		Reference intervals	90%CI (2.5^th^ percentile)	90%CI (97.5^th^ percentile)
ALTnp*	U/L	M^1^		9–50	8–9	50–56
		F^1^		7–37	7–7	35–38
ALTp‡	U/L	M^1^		9–60	9–10	55–63
		F^1^		7–42	7–7	40–44
ASTnp*	U/L	M+F^1^		13–36	13–13	35–37
		M^2^		14–38	14–15	37–39
		F^2^		13–34	13–13	33–35
ASTp‡	U/L	M+F^1^		16–43	15–16	42–45
		M^2^		17–45	17–18	44–47
		F^2^		15–41	15–16	40–42
GGT	U/L	M^1^		10–58	10–10	55–69
		F^1^		7–43	7–8	39–46
ALP	U/L	M^1^		47–123	44–48	121–129
		F^1^	age: 20–49	37–97	33–38	93–101
			age: 50–79	49–134	47–50	128–142
		M+F^2^		39–120	38–40	117–122
Total protein	g/L		region^1^^,^ a	67.9–85.1	67.4–68.3	84.8–85.5
			region^1^^,^ b	70.1–90.1	69.0–70.9	89.9–92.4
		M+F		68.3–87.2	67.9–68.6	86.6–87.7
Albumin	g/L	M+F		43.4–54.6	43.3–43.7	54.4–54.8
Total bilirubin	µmol/L	M^1^		5.9–30.4	5.6–6.1	29.3–33.0
		F^1^		5.0–23.8	4.8–5.3	22.6–25.1
		M+F^2^		5.3–26.8	5.1–5.4	25.6–28.7

1 Reference Intervals with SDR>0.3 as partitioning critical value.

2 Reference Intervals with partition of gender when SDR of gender is 0.2∼0.3; reference Intervals with non-partition of gender when SDR of gender is 0.3∼0.4.

a Reference Intervals for Beijing, Xi'an, Chengdu and Guangzhou.

b Reference Intervals for Shenyang and Shanghai.

* Reagents for analysis of the transaminases without pyridoxal-5′-phosphate.

‡ Reagents for analysis of the transaminases included pyridoxal-5′-phosphate.

We consider that there is no doubt that the partitions are valid under the conditions of SDR<0.2 or >0.4, so for the tests with gender SDR around 0.3 (marginal partition results: SDR is between 0.2–0.4), both gender-specific and pooled reference intervals are listed in Table 3.

### The adjustment and validation of the reference intervals

**Figure 5:**
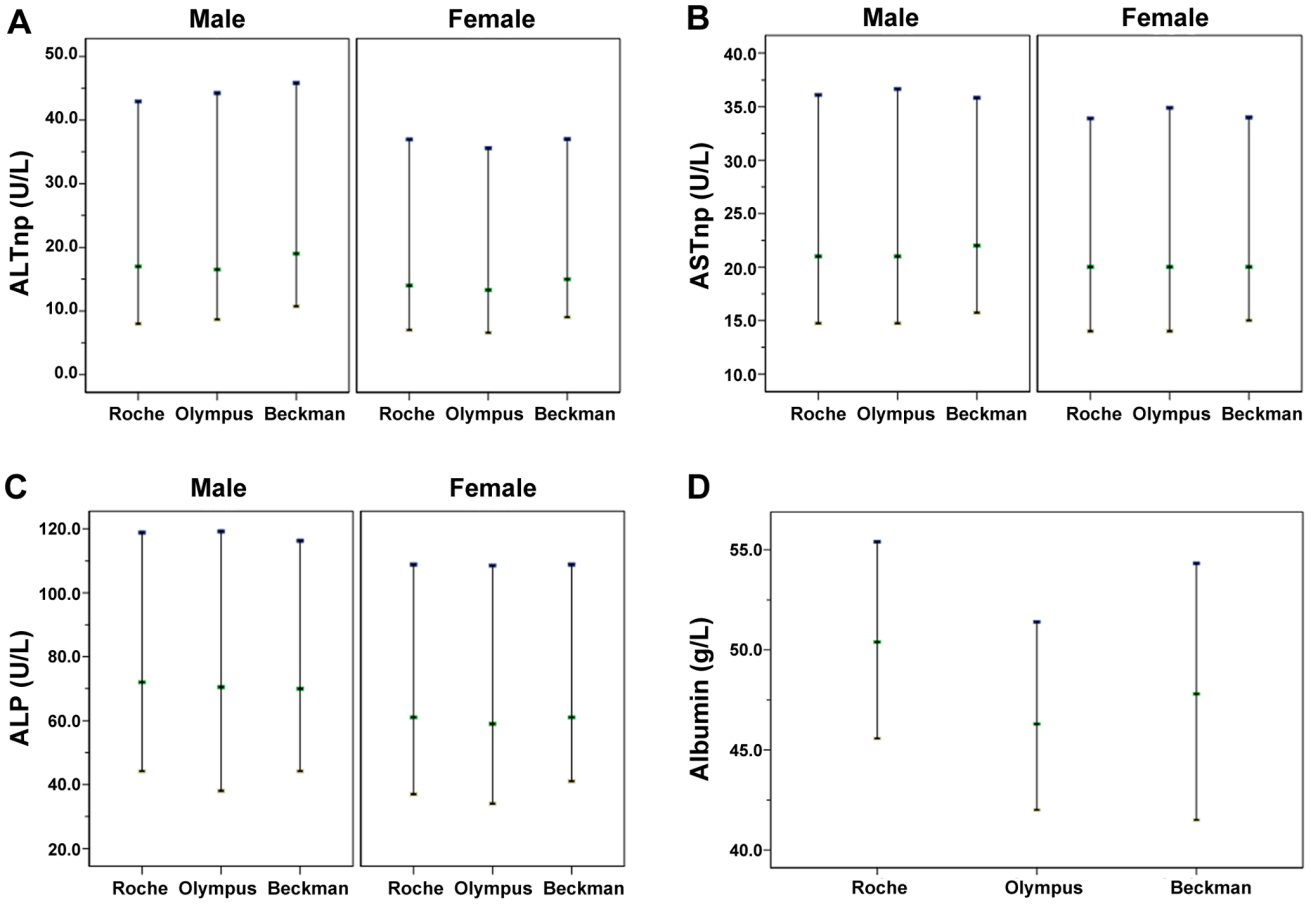
Comparison of the reference intervals of three systems for liver function tests in Shenyang. (A) ALTnp; (B) ASTnp; (C) ALP; (D) Albumin.

For tests whose traceability was not validated in this study (e.g. ALTnp, ASTnp, ALP, ALB), the reference intervals from the three analytical systems (Roche, Olympus, and Beckman system) in Shenyang were compared for consistency (Figure 5). There was a significant difference in the measurement of albumin between the systems (*P*<0.05). In comparison with the data from Roche (BCG method), the median value of albumin from Olympus (BCG method) and Beckman (BCP method) were 4.1 g/L and 2.6 g/L lower, and the lower limits of the reference intervals were 3.6 g/L and 4.1 g/L lower. For the test result of CRM470, Olympus had the highest value, Beckman had an intermediate value, and Roche had the lowest value. The relation of CRM470 results from the three analyzers are completely different from that of human serum results, thus the traceability of albumin results in this study cannot be validated by using CRM470 directly. The final reference intervals of albumin were adjusted according to the results from multiple instruments. For ALTnp, ASTnp and ALP, there were no significant difference among the three systems (*P*>0.05). Thus, the reference intervals of Roche system can be used to represent the three different systems. In addition, for these tests, similar comparisons were also performed for systems in Xi'an and Chengdu and the results were consistent with those of Shenyang.

The reference intervals were used as whole numbers for the convenience of clinicians and further validated in 21 hospitals (Table 4). More than 86% of the 21 laboratories passed the validation in each subgroup of reference intervals and overall about 95.3% to 98.8% of the 1220 validation data fell within the range of the reference intervals for all liver function tests. The validation results suggest that the adjusted reference intervals are applicable in most of the qualified laboratories in China.

**Table 4:** The results of reference interval validation in 21 laboratories.

Analytes	Unit	Partition	Reference Intervals after adjustment	Number of validation labs	Labs with open system	Number of acceptable labs	Percentage of acceptable labs	Number of validation Samples	Percentage of acceptable samples*
ALTnp	U/L	M	9–50	21	12 (57.1%)	20	95%	482	97.5%
	U/L	F	7–40	21	12 (57.1%)	21	100%	738	98.0%
ASTnp	U/L	M	15–40	21	11 (52.4%)	20	95%	482	96.9%
	U/L	F	13–35	21	11 (52.4%)	21	100%	738	97.8%
GGT	U/L	M	10–60	21	12 (57.1%)	21	100%	482	97.3%
	U/L	F	7–45	21	12 (57.1%)	20	95%	738	96.7%
ALP	U/L	M	45–125	21	10 (47.6%)	21	100%	482	97.1%
	U/L	F(20–49 years)	35–100	21	10 (47.6%)	18	86%	467	95.3%
	U/L	F(50–79 years)	50–135	21	10 (47.6%)	20	95%	411	95.6%
Total protein	g/L	M+F	65–85	21	11 (52.4%)	21	100%	1220	98.7%
Albumin	g/L	M+F	40–55	21	11 (52.4%)	20	95%	1220	98.8%
Total bilirubin	µmol/L	M	6–30	21	13 (61.9%)	19	90%	482	97.1%
	µmol/L	F	5–24	21	13 (61.9%)	19	90%	738	95.7%

* The percentage of results of validation samples inside the reference intervals of this study.

### Comparison of reference intervals

In comparison with the reference intervals obtained from this study, the reference limits and partitioning of reference intervals from different sources (Asians (Chinese), Whites, Blacks or multi-racial people) (Table 5) for analytes such as ALTp, ALP, total bilirubin were different. In addition, we evaluated the applicability of the reference intervals recommended by the Chinese National guide to Clinical Laboratory Procedures (third edition), and found that some of those intervals do not reflect the current Chinese population. For example, the single side observed proportions of out of range (OOR) of reference values from our study for most of the tests deviated significantly from the nominal 2.5% such as total bilirubin (15.2%), ALTp (Male: 9.7%, Female: 10.8%), ALP (0.2%), albumin (0.0%).

**Table 5:** Comparison of established reference intervals in this study against various sources.

	This study	Chan et al [11]	Ceriotti et al [8]	Rustad et al [6]	Karita et al [7]	National guide to Clinical Laboratory Procedures (3rd Ed.)^1^ [2]	OOR*(%)
Population	Chinese	Chinese	European & Asian	European	African		
Region	6 regions in China	Hong Kong, China	Italy, China, North Europe & Turkey	5 countries in North Europe	4 countries in East & South Africa		
Multi-center	Yes	No	Yes	Yes	Yes		
Number of samples							
M+F	3210	138	765	3036	2105		
M	1274	197	354	1427	1083		
F	1936	335	411	1609	1022		
Age	20–79	21–81	18–90	≥18	18–60		
Specimen type	Serum	Serum	Serum	Serum/Plasma	Serum		
Partition Method	Nested ANOVA	Harris & Boyd	Lahti	Lahti	ANOVA		
Calculation of reference interval	Nonparametric method	Nonparametric method	Nonparametric method	Nonparametric method	Nonparametric method		
**Analytes**							
ALTp(U/L)							
M+F					8–61		
M	9–60		9.0–59.0	10–68		13–40	9.7%
F	7–45		7.8–41.0	8–46		10–28	10.8%
ALTnp(U/L)							
M	9–50	13–53				5–40	6.5%
F	7–40	8–36				5–35	3.0%
ASTp(U/L)							
M+F			11–34		14–60	8–40	3.8%
M	15–45			14–45			
F	13–40			13–37			
ASTnp(U/L)							
M	15–40					13–40	1.4%
F	13–35					10–28	8.0%
GGT(U/L)							
M	10–60		11.7–67.5	18–39years:12–78; ≥40years:15–114		11–50	4.7%
F	7–45		6.4–39.7	18–39years:10–42; ≥40years:11–77		7–32	5.6%
ALP(U/L)							
M+F				37–106	48–164	40–150	0.2%
M	45–125	39–97					
F	20–49years:35–100; 50–79years:50–135	34–97					
Total protein(g/L)							
M+F	65.0–85.0	66–80		62.4–77.9	58–88	64–83	0.3%
Albumin(g/L)							
M+F	40.0–55.0	39–50		18–39years:36.5–47.9; ≥40years:36.5–45.4	35–52	34–48	0.0%
Total bilirubin (µmol/L)							
M+F				4.7–24.0	2.9–37.0	3.4–17.1	15.2%
M	6.0–30.0	6–32					
F	5.0–24.0	5–27					

1 Currently recommended reference intervals in China.

* OOR (out of range): The single side observed proportions of reference values in this study outside the reference intervals recommended by National guide to Clinical Laboratory Procedures (third edition). OOR for total protein and albumin: the percentage less than lower limit; OOR for ALTp, ALTnp, ASTp, ASTnp, GGT, ALP and total bilirubin: the percentage over upper limit.

## Discussion

Our research fills the gap of Chinese-specific reference intervals for liver function tests. The definition of the reference collective, the number of individuals enrolled, the traceability of tests, and the statistical analysis methods used in this study met the requirements set by Ceriotti for establishing common reference intervals [5]. For the reference interval establishment, of all the subgroups in our study, the least number of reference individuals was from the subgroup of female and 50–79 years old for ALP, which is 932, far more than 400, which ensures a high accuracy of the reference interval with narrow confidence intervals on each of the derived reference limits [18].

The quality of reference interval depends on the appropriate reference individual choice. Individuals with common diseases that affect levels of liver function tests were excluded by specific screening procedure such as metabolic syndrome and fatty liver disease. In recent years, the number of people with metabolic syndrome (MS) has increased rapidly, and the studies of Marchesini et al and Kim et al found that ALT, AST and GGT values are significantly enhanced in an MS group as compared with a control group [19], [20]. For this reason, we excluded all individuals who met the metabolic syndrome diagnostic criteria. The prevalence of FLD (including alcoholic fatty liver disease (AFLD) and non-alcoholic fatty liver disease (NAFLD)) is increasing globally, and some studies have already shown that more patients with FLD are found in various regions and ethnic groups across China [21], [22]. There is a strong positive correlation between NAFLD and age, BMI, cholesterol, and glucose [23]. Since all these factors may lead to a significant increase of liver enzyme levels [20], [24], [25], we investigated the influences of the individuals with FLD on the establishment of reference intervals after the screening in our study. 96 individuals with ALT≥30 U/L were followed and indeed 11 patients out of these suffered from fatty liver disease as shown with ultrasonography. Within these 11 patients, 2 males and 1 female had ALTnp values above the upper reference limit described in this study, that is, the percentage of FLD-individuals with ALTnp values above the upper reference limit in this study is about 0.33%. With similar calculation of ALTnp mentioned above, we estimated that the percentage of FLD-individuals with GGT and ALP values above the upper reference limit is lower than ALTnp. Therefore it is reasonable to conclude that the effect of fatty liver disease on the upper limit of liver enzyme reference is less significant after the screening, especially for BMI, triglycerides, total cholesterol, glucose.

The traceability of ALTnp, ASTnp, ALP, albumin cannot be validated in this study. For albumin, we tried to use CRM470 to evaluate the trueness and found that this reference material is not commutable between the three systems or two methods. This may possibly be due to the matrix effect of CRM470, difference of assay reagents or specificity of assay methods. Therefore, it is yet impossible to use CRM470 to evaluate the trueness of albumin test results from the three systems directly. In 2011, ALP reference method recommended by IFCC had been established. But it is yet impossible to evaluate the ALP results because there is no ALP reference material available currently. We used a pooled human serum with the target-value assigned by a number of joint reference laboratories in China for preliminary evaluation of the results obtained using Roche Modular analyzers, and found that the ALP results were about 10% lower. For ALT and AST detection, most laboratories in China only use reagents without pyridoxal-5′-phosphate because of the better reagent stability. The upper reference limits obtained from reagents without pyridoxal-5′-phosphate are about 10% lower than those from pyridoxal-5′-phosphate reagents. For the four laboratory tests mentioned above, consensus reference intervals were determined according to the results from multiple instruments.

It has been shown that the upper reference limits of liver enzymes such as ALT, AST, GGT and ALP described in the literatures are quite different. First of all, this may be due to race difference, as shown in Dufour's study that the AST activity of African-American males is 15% higher than that of the other races of American males. ALP level of the blacks is generally 10–15% higher than that of the Whites [26]. As shown in Table 5, we found that the ASTp and ALP results from southeast Africa are significantly higher than that from other races. In addition, the method for selecting reference individuals may be another contributing factor. The screening criteria and the defined conditions for the same criteria are usually different for different studies. Ceriotti et al [8] and Chan [11] used multiple test parameters as the screening criteria for participants. These parameters include BMI, triglycerides, total cholesterol, glucose etc which posed relatively heavy influence on liver enzyme. In the Nordic and African studies, no reference individual screening with similar laboratory test parameters were conducted. In our study, we have set the limitation of BMI, triglycerides, total cholesterol, and glucose for reference intervals screening and, in addition, excluded certain individuals with NAFLD and MS. Selection of different screening criteria may lead to different results.

Some studies have shown that the postmenopausal hormone level changes could up-regulate the bone metabolism to a high conversion status with increased bone formation and bone absorption [27]. This may be the main reason for the much higher ALP reference interval for the female older than 50 years in our study.

In agreement with other studies [28], [29], our study showed that the albumin level gradually decreases with age which makes it difficult to define a clear age cut-off point for albumin. So our study provided a unified reference interval of albumin which had no significant deviation caused by sampling procedure of this study and was applicable to the current demographic distribution in China because it shows no significant difference from the age-standardized reference intervals. The observed lower albumin reference limits from different regions, age, and urban-rural groups detected by different analytical systems or methods, and the results are all found above 40 g/L and are significantly higher than the current, well-accepted value of 35 g/L for the European, American and African populations. This may be related to the Chinese dietary patterns with high intake of soy products containing high amount of plant protein. Compared with the results from China National Nutrition and Health survey in 2002 [30], albumin level of participants in this study is higher on average and the proportion of population with low serum albumin level decreased significantly. It indicates that the malnutrition situation of Chinese people has been improved to certain level because of the increasing protein intake thanks to the rapid economic growth in China during the past decade. Our results confirmed that the albumin reference intervals of previous survey do not apply to the current population.

The total bilirubin values for the Chinese population in our study are much higher than those of the European and American populations, but significantly lower than that of the African populations. In this study, some young healthy individuals had significantly higher total bilirubin levels. This may possibly caused by congenital hemolytic jaundice (Gilbert's syndrome), thus could further affect the upper limits of the reference intervals. The mechanism for this elevated total bilirubin levels is not fully clear and needs further investigation. Compared with other studies, the total bilirubin results of the Hong Kong populations are quite similar to those of our study.

The proportion of OOR values of most liver function tests deviate significantly from 2.5% if we use the reference intervals in National Guide to Clinical Laboratory Procedures (third edition). Therefore, we can conclude that these used reference intervals are no longer applicable for the current Chinese population. The reason is firstly because the reference intervals listed in the National Guide to Clinical Laboratory Procedures (third edition) were mainly established with European and American populations by different study approaches or different detection methods. Secondly, the lifestyle, diet and nutritional status of the current populations have changed dramatically due to the continuous improvement of the economic condition and this had a significant impact on the reference intervals.

As a large developing country, China has still limited resource for health care. Using international analytical systems for patient sample assay usually induces high cost. Therefore, many hospitals in China use open-systems to reduce the overall cost. Most of 21 labs (half of them are using open-system) passed the reference interval validation for each analyte in our study. This implies that: first of all, the reference intervals established in our study are applicable for most of the population in the nation. Secondly, regardless of using open systems or close systems, the reference intervals established in our study can be used directly by most of the hospitals with guaranteed accuracy.

Since the results of validation in our study confirmed the reference intervals are applicable for the Chinese population across the country. However, in addition to Han, there are also a number of different ethnic in China (accounting for 8.4% of the total population), who have not been covered yet by our study so far. We have already started to recruit reference individuals from several large ethnic populations (such as Korean, Tibetan) in China for preliminary assessment, and we plan to complete the establishment of the reference intervals for the major ethnic populations in China in the near future. This study explores the study method and application evaluation mode of the reference interval suitable for China's current situation. Reference intervals of liver function tests of our study could provide appropriate laboratory basis to contribute to the diagnosis, treatment and monitoring of disease, and help to conserve healthcare resources and reduce overall healthcare cost.

## Conclusions

The used reference intervals of liver function tests are no longer applicable for the current Chinese population. Our study filled the critical gap that there had not been a multi-center based study with a large number of samples for defining Chinese-specific reference intervals. We have established accurate, applicable, and common reference intervals of liver function tests for the Chinese adult population.
